# Combining chemotherapy with CAR-T cell therapy in treating solid tumors

**DOI:** 10.3389/fimmu.2023.1140541

**Published:** 2023-03-06

**Authors:** Arthur Xuan Wang, Xiao Jing Ong, Criselle D’Souza, Paul J. Neeson, Joe Jiang Zhu

**Affiliations:** ^1^Cancer Immunology Program, Peter MacCallum Cancer Centre, Melbourne, VIC, Australia; ^2^Sir Peter MacCallum Department of Oncology, Faculty of Medicine, Dentistry and Health Science, University of Melbourne, Melbourne, VIC, Australia

**Keywords:** chemotherapy, Chimeric Antigen Receptor T cell (CAR-T), solid tumor, tumor microenvironment (TME), personalized combination

## Abstract

Chemotherapy has long been a standard treatment for a wide range of malignancies, where patients typically undergo multiple rounds of chemotherapy regimens to control tumor growth. In the clinic, the chemotherapy drugs cyclophosphamide and fludarabine are commonly used prior to Chimeric Antigen Receptor T (CAR-T) cell therapy to lymphodeplete and improve CAR-T cell engraftment. In this review, we discuss the use of chemotherapy in combination with CAR-T cell therapy. We also show that chemotherapy can deplete immunosuppressive cells, promote a pro-inflammatory tumor microenvironment, disrupt tumor stroma, and improve CAR-T cell recruitment to the tumor. Although the combination of chemotherapy plus CAR-T cell therapy is promising, certain aspects of chemotherapy also pose a challenge. In addition, the combined therapeutic effect may be heavily dependent on the dose and the treatment schedule. Thus, we also discussed the obstacles to effective clinical outcomes of the combination therapy.

## 1 Introduction

Recently, a large number of cancer therapies have been developed to facilitate a patient’s immune system against cancers. One such therapy is called the Chimeric Antigen Receptor (CAR) T cell therapy, which involves the adoptive transfer of autologous T cells that have been genetically engineered with a CAR to target tumor cells (1). CAR-T cell therapy has induced remission in patients with relapsed/refractory B-cell malignancies (2–4). However, this success has not occurred in patients with solid tumors. The reasons for this poor outcome include tumor heterogeneity, an immunosuppressive tumor microenvironment (TME), insufficient T-cell trafficking to the tumor site, and poor T-cell persistence (5).

Chemotherapy has long been a standard-of-care treatment for many cancers, especially advanced solid tumors. The successful treatment of cancers requires a combination of different approaches. However, because chemotherapy can exert negative effects on the immune system, it is not clear whether chemotherapy can be combined with immunotherapy more broadly. Indeed, different classes of chemotherapeutic drugs such as alkylating agents (e.g., cyclophosphamide), platinum compounds (e.g., cisplatin, carboplatin, oxaliplatin), antimetabolites (e.g., methotrexate), anthracyclines, DNA methyltransferase inhibitors and spindle poisons (e.g., taxanes) have mixed effects on the immune system (6). Certain drugs can induce profound immunosuppression, while some drugs can enhance anti-tumor immunity. Some synergistic effects include relieving tumor-induced immunosuppression, augmenting the anti-tumor activity of cytotoxic immune cells, and improving immune cell trafficking to tumor sites. As a result, these positive effects of chemotherapy could overcome some of the roadblocks for CAR-T cell therapy in treating solid cancers. Hence, this review will summarise the main effects of chemotherapy when combined with CAR-T cells, including the promises and challenges of combination therapy for solid cancers.

## 2 How can chemotherapy be used in conjunction with CAR-T cell therapy?

CAR-T cell therapy have produced impressive clinical responses in relapsed/refractory B cell malignancies. However, there are numerous factors to be considered during the treatment, including the need for a bridging therapy or conditioning regimen prior to the infusion of CAR-T cell product. These regimens significantly impact the clinical outcomes.

### 2.1 Chemotherapy as a bridging therapy

The infusion of CAR-T cells must be performed in a timely manner to control disease progression (7). However, the period between apheresis and CAR-T cell infusion can be weeks to months, e.g. a maximum of 105 days (median time of 45 days) in the ELIANA trial (NCT02435849) (3). This time interval may lead to a treatment gap where some patients may experience disease progression and/or death. As a result, 7% of patients did not survive while awaiting the production of CAR-T cells (8, 9). Therefore, bridging therapy is critical in controlling disease burden prior to CAR-T cell treatment.

The choice of bridging therapy is highly variable, depending on the patient’s cancer type, disease stage, prior treatments, and disease burden. Chemotherapy is one of the most widely used bridging therapies, including kinase modulators, topoisomerase inhibitors, platinum-based agents, and drugs that interfere with DNA replication, synthesis and repair (10). These drugs can inhibit tumor growth to achieve disease control in the bridging period. As a bridging therapy, chemotherapy can also debulk the tumor before CAR-T cell infusion in some cases. This is an important consideration as high disease burdens have been associated with toxicities following CAR-T cell infusions, such as cytokine release syndrome (CRS) and immune effector cell-associated neurotoxicity syndrome (ICANS) (11).

### 2.2 Chemotherapy as a conditioning regimen

Studies showed that lymphodepletion prior to adoptive T-cell transfer significantly enhanced their expansion, engraftment, and anti-tumor efficacy (12). This enhancement is likely due to reduced immunosuppressive cells (e.g., myeloid-derived suppressor cells and regulatory T cells) (12, 13), increased homeostatic cytokine production (14, 15), and the downregulation of indoleamine 2,3-dioxygenase (IDO) expression in the tumor (16). Together, these mechanisms may create an optimal environment for the anti-tumor function of infused CAR-T cells. Along with improved CAR-T cell engraftment and homeostatic expansion, these findings led to the introduction of lymphodepletion conditioning regimens with CAR-T cell therapy.

Chemotherapeutic drugs such as cyclophosphamide (Cy) and fludarabine (Flu) are the most commonly used lymphodepleting regimen in CAR-T cell therapy (250-500 mg/m^2^ cyclophosphamide, 25-30 mg/m^2^ fludarabine, 3-5 days before infusion) (17, 18). Compared to Cy alone, the combination of Cy/Flu significantly enhanced CAR-T cell expansion and was associated with better clinical responses (Cy/Flu = 50% CR, 72% ORR versus Cy alone = 8% CR, 50% ORR) (19). In the JULIET trial, patients with the Cy-resistant disease also received bendamustine (90 mg/m^2^, 2-11 days before infusion) in lieu of Cy/Flu, with similar clinical outcomes (20). However, there are risks associated with intense lymphodepletion, including prolonged neutropenia and toxicities. Therefore, further studies are needed to investigate the favourable dose and schedule of lymphodepletion with minimal side effects and enhanced clinical response to CAR-T therapy.

### 2.3 Chemotherapy as a neoadjuvant/adjuvant treatment

Although chemotherapy has not been conventionally utilized as a neoadjuvant or adjuvant treatment for CAR-T cell therapy, emerging evidence suggests that specific chemotherapy drugs may work in concert with CAR-T cells (6, 21) (**Table 1**). This novel combination strategy shows great potential to overcome some barriers in CAR-T cell therapy and provide synergistic effects in treating solid tumors.

**Table 1:** Chemotherapeutic drugs that can be used as neoadjuvant or adjuvant regimen with CAR-T cell therapy - preclinical and clinical evidence.

Immunomodulatory effects of chemotherapeutic agents							
Drug class	Agent		Cancer type/Animal model		Effect		Reference
Alkylating agents	Cyclophosphamide		Pleural mesothelioma		Treg depletion and expansion of CD8^+^ T_EM_ cells.		(22)
			Metastatic solid tumors		Treg depletion and restoration of T-cell function.		(23)
			Metastatic breast cancer		Treg depletion and expansion of tumor-specific T cells.		(24)
			Metastatic colorectal cancer		Reduced proportions of Tregs, B cells and NK cells. Expansion of tumor-specific T cells.		(25)
	Dacarbazine		Melanoma		Increased T-cell recruiting chemokine production.		(26)
			Stage III/IV melanoma		Increased chemokine production and ECM remodelling.		(27)
	Temozolomide		Murine glioma model		Treg depletion and inhibition of Treg function.		(28)
			Advanced melanoma		Depletion of Tregs.		(29)
			Human/murine melanoma model		Increased T-cell recruiting chemokine production and T-cell infiltration.		(26)
Anti-metabolites	Gemcitabine		Pancreatic ductal adenocarcinoma		Depletion of granulocytic MDSCs and Tregs in the peripheral blood and reduction of plasma TGFβ-1 levels.		(30)
			Pancreatic ductal adenocarcinoma		Polarisation of TAMs into M1-like phenotype and induced secretion of immunostimulatory cytokines.		(31)
			Various murine cancers		Depletion of MDSCs and inhibition of MDSC function.		(32)
	Gemcitabine and 5-fluorouracil		Pancreatic and oesophageal cancer		Depletion of MDSCs in peripheral blood.		(33)
			Murine thymoma model		Depletion of MDSCs in spleen and at tumor site.		(34)
Anthracyclines	Doxorubicin		Murine mammary cancer		Depletion of MDSCs and inhibition of MDSC function.		(35)
Taxanes	Docetaxel		NSCLC patients		Depletion of Tregs in the peripheral blood.		(36)
			Murine mammary tumor model		Inhibition of MDSC function and restoration of T cell function. Polarisation of MDSCs into M1-like phenotype.		(37)
	Paclitaxel		Advanced non-small cell lung cancer		Depletion of Tregs and inhibition of Treg function.		(38)
			Murine orthotopic renal cell carcinoma model		Depletion of Tregs and inhibition of Treg function.		(39)
			Murine spontaneous melanoma model		Depletion of MDSCs and inhibition of MDSC function.		(40)
			Murine breast cancer and melanoma models		Polarisation of TAMs into M1-like phenotype.		(41)
			Human pancreatic cancer xenograft models		Depletion of tumor stroma.		(42)
			Advanced pancreatic cancer		Depletion of CAFs and disruption of tumor ECM.		(43)
Platinum compounds	Carboplatin, cisplatin and oxaliplatin		Melanoma and colorectal cancer		Downregulation of PD-L1/L2 on tumor and dendritic cells.		(44)
	Carboplatin + anthracyclines and taxanes		Human breast cancer		Increased T-cell recruiting chemokine production.		(45)
	Carboplatin		Human NSCLC cell lines and murine Lewis lung carcinoma mouse model		Increased T-cell recruiting chemokine production and infiltration of CD8^+^ T cells.		(46)
	Oxaliplatin + cyclophosphamide		Murine lung adenocarcinoma models		Increased T-cell infiltration through immunogenic tumor cell death. Increased CD8^+^ T cells:Treg ratio at tumor.		(47)
Combination with CAR-T cells							
Drug combination		Cancer type/Animal model		Outcome		Reference	
Cyclophosphamide pre-conditioning + BCMA-CAR T cells		Phase I trial for multiple myeloma (MM) patients		7 out of 11 patients achieved partial response (PR) or better.		NCT02546167 (48)	
Cyclophosphamide pre-conditioning + CD19-CAR T cells		Phase I trial for chemotherapy-refractory chronic lymphocytic leukaemia (CLL) & Relapsed B-cell acute lymphoblastic leukaemia (B-ALL)		2 out of 5 patients with this combination achieved stable disease (SD).		NCT00466531, NCT01044069 (49)	
Cyclophosphamide pre-conditioning + PSCA-CAR T cells		Human PSCA knock-in mice bearing subcutaneous human PSCA tumors		Induced complete responses (CR) in 3/7 mice. Increased CAR-T cell infiltration into TME. Polarisation of TME into pro-inflammatory state. Reduced M2-like myeloid cells.		(50)	
Cyclophosphamide/ Fludarabine pre-conditioning + CD19-CAR T cells		Phase I/II for relapsed/refractory CLL, NHL and ALL		16 out of 17 patients achieved complete response (CR).		NCT01865617 (51)	
Cyclophosphamide/Fludarabine pre-conditioning + CD19-CAR T cells		Murine subcutaneous lymphoma xenograft model		Downregulation of IDO expression in lymphoma cell lines and increased efficacy of CAR T-cells against tumors.		(16)	
Cyclophosphamide/Fludarabine pre-conditioning + GD2-CAR T cells		Phase I relapsed/refractory neuroblastoma		Increased CAR T cell expansion and serum IL-15 levels. Greater patient survival (6 out of 7).		NCT01822652 (52)	
Cyclophosphamide/Fludarabine pre-conditioning + CD19-CAR T cells		Phase I/II trial for advanced-stage B cell lymphoma		Increased serum IL-15 levels. 12 out of 22 patients achieved complete response (CR) and 4 achieved partial remission (PR).		NCT00924326 (15)	
Docetaxel + PSMA-CAR T cells		Human prostate cancer subcutaneous xenograft model		Remodelling of TME by inducing tumor damage and altering tumor stroma. Increased infiltration of CAR-T cells Reduction of tumor growth.		(53)	
Docetaxel adjuvant + PSMA-CAR T cells		Human prostate cancer liver metastasis mouse model & human prostate cancer xenograft mouse model		Reduction of exhaustion markers (PD-1, TIM3, CTLA3) on CAR T cells. Depletion of MDSCs in peripheral blood.		(54)	
Doxorubicin + systemic IL-2 + CD19-CAR T cells		Various murine and human xenograft solid tumors		Increased infiltration of CAR T-cells into tumor. Reduction in tumor infiltrating Tregs. Suppression of co-inhibitory receptor (PD-1 & LAG3) expression on T cells. Promotes expression of T-cell recruiting chemokines in tumor cells (CXCL9, CXCL10) and CXCR3 receptors on CAR T cells.		(55)	
Doxorubicin pre-treatment + GD2-CAR T cells		Osteosarcoma cell lines		Decreased PD-L1 expression on tumor cells.		(56)	
Gemcitabine pre-treatment + CD19-CAR T cells		Phase I/IIa trial for CD19-positive B-cell lymphoma or leukaemia		3 out of 5 patients achieved complete response (CR) and 1 achieved SD.		NCT02132624 (57)	
Low dose cyclophosphamide pre-conditioning + Ig kappa (κ)-CAR-T cells		Phase I trial for non-Hodgkin lymphoma/chronic lymphocytic leukaemia (NHL/CLL) & Multiple Myeloma (MM)		4 out of 9 NHL/CLL and 4 out of 7 MM patients achieved clinical response.		NCT00881920 (58)	
Nab-paclitaxel/Cyclophosphamide/Fludarabine pre-conditioning + Claudin18.1-CAR T cells		Phase I trial for gastric cancers		Achieved responses in 21/28 patients who had previously failed taxane treatment.		NCT03874897 (59)	
Oxaliplatin + Cyclophosphamide + ROR1-CAR T cells		Murine metastatic transplantable lung adenocarcinoma model		Polarisation of TME into pro-inflammatory state. Promoting the expression of T-cell recruiting chemokines in tumor-infiltrating macrophages. Increased CAR-T cell infiltration into TME.		(60)	
Temozolomide pre-conditioning + EGFRvIII-CAR T cells		Murine glioblastoma models		Increased CAR-T:Treg ratio in the tumors. Increased CAR-T cell expansion and persistence.		(61)	

The immunosuppressive tumor microenvironment (TME) in solid tumors is a significant obstacle to achieving clinical response to CAR-T cell therapy (62). Numerous immune suppressive cells infiltrate the TME, including myeloid-derived suppressor cells (MDSCs), tumor-associated macrophages (TAMs), and regulatory T cells (Tregs). These cells promote immunosuppression and negatively regulate CAR-T cell effector function (63). In addition, cancer-associated fibroblasts (CAFs) produce extracellular matrix components in the TME (64), forming a physical barrier restricting CAR-T cell penetration. Together, these cells play a pro-tumorigenic role that interferes with CAR-T cell efficacy (65–67). This anti-inflammatory milieu would lead to poor CAR-T cell penetration, expansion, and persistence, rendering the therapy ineffective.

In this regard, chemotherapy is a promising approach for remodelling the TME, and could lead to the enhanced therapeutic efficacy of CAR-T cells in solid tumors. Chemotherapy drugs can modify the TME in four main ways: reducing immune suppressor cells, repolarising the anti-inflammatory immune microenvironment, disrupting the tumor stroma, and altering the chemokine profile for T cell trafficking. These mechanisms are discussed in the following sections of this review.

#### 2.3.1 Chemotherapy reduces immune suppressor cells in the TME

**Figure 1:**
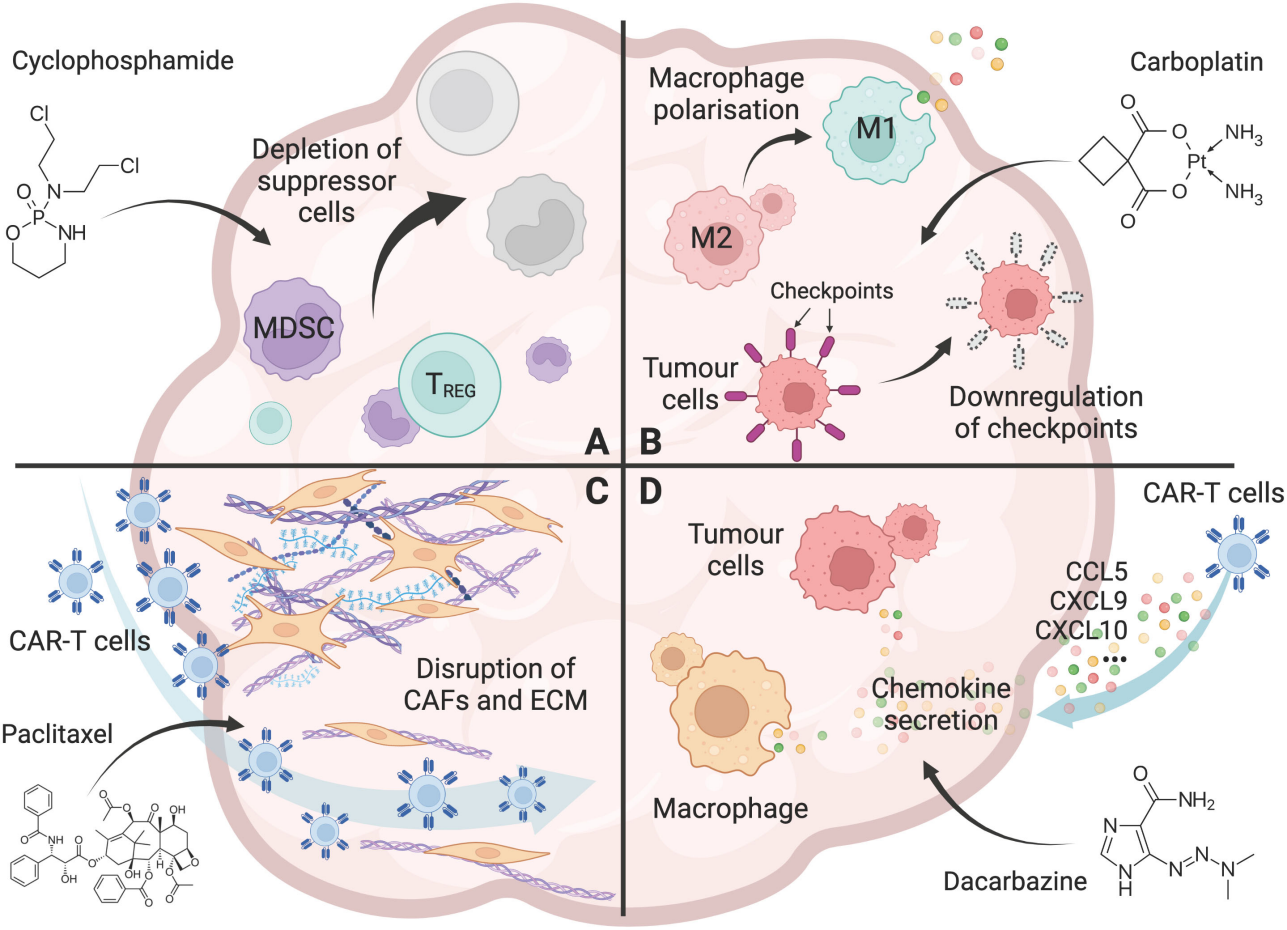
Chemotherapy overcomes the hurdles for CAR-T cells in solid tumors. **(A)** The effect of chemotherapy (Cyclophosphamide as an example) on the depletion of immunosuppressive cells, including Tregs, tumor associated macrophages (TAMs), and myeloid-derived suppressor cells (MDSCs) in the tumor microenvironment. **(B)** The effect of chemotherapy (Carboplatin as an example) on promoting a pro-inflammatory tumor microenvironment, including polarisation of macrophages from an M2 to an M1 phenotype, and downregulation of immune checkpoint molecules including PD-L1 and PD-L2 on tumor cells. **(C)** The effect of chemotherapy (Paclitaxel as an example) on the disruption of the tumor stroma, including removal of cancer-associated fibroblasts (CAFs) and their associated extracellular matrix (ECM) from the tumor barrier, allowing increased T-cell infiltration into the tumor. **(D)** The effect of chemotherapy (Dacarbazine as an example) on enhanced T-cell trafficking to the tumor, including the increased chemokine CCL5, CXCL9, and CXCL10 secretion by tumor cells and macrophages. Created with BioRender.com.

Chemotherapy has been shown to reduce the various immune suppressor cells in the TME. Cy/Flu conditioning regimen have enhanced CAR-T cell expansion and clinical responses in cancer patients (48, 49, 51, 52). Several preclinical and clinical studies have shown that metronomic, low-dose Cy regimen (12.5mg/kg, single dose) reduced the number and function of Tregs, thereby restoring tumor-specific T cell responses (22–25, 58, 68) (**Figure 1A**). Importantly, low-dose Cy does not affect tumor-responding T cells (24), suggesting that low-dose Cy could be used as an adjuvant treatment post-CAR-T cell infusion.

Temozolomide is another potential neoadjuvant treatment for patients with glioblastoma receiving CAR-T cells. Temozolomide reduced Tregs in both mouse models and in patients (28, 29). In murine glioblastoma models, temozolomide treatment also increased CAR-T cell expansion resulting in a greater CAR-T cell to Treg ratio (61).

Two taxane drugs (paclitaxel and docetaxel) reduced immune suppressor cells in the TME. Tregs in non-small cell lung cancer patients were reduced after four cycles of docetaxel (30 mg/m^2^) and cisplatin (75 mg/m^2^) (36). A similar reduction in Tregs and impairment of their inhibitory function were also observed with paclitaxel treatment (38, 39). In addition, when given as neoadjuvant treatment prior to CAR-T cell infusion, docetaxel was found to enhance the PSMA-CAR-T cell efficacy in prostate cancer mouse models, by reducing MDSCs in the tumor (54). Similarly, paclitaxel has also been found to reduce the tumor-infiltrating MDSCs and restore effector functions of CD8^+^ T cells (40). However, this impact was not consistently observed across other studies (34, 69). This discrepancy could be due to differences in drug dosage and treatment schedule.

Gemcitabine can also deplete MDSCs in cancer patients. In a Phase I/IIa trial of CAR-T cell therapy, three out of five patients who received gemcitabine as neoadjuvant treatment achieved complete response (CR) following CAR-T infusion (57). Preclinical and clinical studies demonstrated that gemcitabine treatment reduced the amount of MDSCs (30, 33) and increased the cytotoxicity of anti-tumor CD8^+^ T cells and NK cells (32), this may contribute to enhanced responses to CAR-T cells.

Doxorubicin is another chemotherapeutic drug shown to reduce Tregs and MDSCs in the TME. When combined with CAR-T cells (55) or adoptively transferred T-helper cells (35), doxorubicin synergistically increased anti-tumor activity by diminishing the Tregs and MDSCs, leading to tumor suppression in both murine and human xenograft models.

#### 2.3.2 Chemotherapy polarizes the TME

In solid tumors, tumor cells can express high levels of immune checkpoint molecules, including PD-L1 and PD-L2, to inhibit the effector functions of tumor-infiltrating T cells and CAR-T cells (70). In this regard, doxorubicin has been shown to affect CAR-T cell activity by lowering the expression of the immune checkpoint PD-L1 on tumor cells in osteosarcomas (56). Platinum-based chemotherapeutic drugs such as carboplatin, cisplatin, and oxaliplatin have also been shown to reduce PD-L2 expression on tumor cells, resulting in increased tumor cell recognition, proliferation and cytokine secretion of tumor-specific T cells (44) (**Figure 1B**).

Studies also revealed that oxaliplatin ameliorates the anti-inflammatory TME and enhances the recruitment of ROR1-CAR-T cells through modulating the TME chemokine profile (60). This polarisation of anti-inflammatory TME into a favourable pro-inflammatory state was also observed in CAR-T cell therapy with prior Cy treatment (50). The change in TME polarisation into a more inflammatory milieu is most likely accomplished through the activation and differentiation of myeloid cells into a pro-inflammatory phenotype. This TME change produces chemokines that recruit CAR-T cells, transforming the “cold” tumor into a “hot” tumor. In addition, paclitaxel, docetaxel, and gemcitabine were also shown to polarise macrophages into an M1-like phenotype to facilitate anti-tumor immunity (31, 37, 41, 71). This further provides a rationale for the combination therapy of CAR-T cells with chemotherapeutic drugs to overcome the immunosuppression in solid tumors.

#### 2.3.3 Chemotherapy disrupts the tumor stroma

Besides the suppressor cells, the immunosuppressive tumor microenvironment is also enriched with stroma that could exclude CAR-T cell infiltration. The anti-microtubule agent, nab-paclitaxel, damaged the tumor stroma in primary tumors of advanced-stage prostate cancer patients (42, 43). Patients treated with nab-paclitaxel demonstrated less abundant fibrillar collagen matrices and lower CAF numbers, which were also confirmed using mice patient-derived xenograft (PDX) models (43). In a Phase I clinical trial of combination treatment of Claudin18.2-CAR-T cells with paclitaxel plus Cy, 21 out of 28 patients who failed prior taxane treatment achieved a significant clinical response to the combination treatment. The author suggested that this effect may be due to the accumulated nab-paclitaxel in the tumor stroma, which disrupted cancer-stromal interactions and helped with the CAR-T cell infiltration (59) (**Figure 1C**). Similarly, in a human xenograft mouse model, it was found that PSMA-CAR-T cells, in combination with low-dose, non-ablative docetaxel as a neoadjuvant regimen, eradicated large established tumors (53). Further analysis revealed that docetaxel could remodel the TME by altering the tumor stroma, which allowed the infiltration of CAR-T cells into the tumor site. These findings raise the possibility of using chemotherapy to overcome the physical barrier of TME, allowing CAR-T cell penetration.

#### 2.3.4 Chemotherapy enhances T cell trafficking to tumors

T-cell trafficking to tumors remains a significant challenge for CAR-T cell efficacy. Successful T-cell trafficking depends on various chemokines including CCL5, CXCL9 and CXCL10 (72), and is critical for the response to CAR-T cell therapy. In cutaneous melanoma mouse models, temozolomide increased the expression of CCL5, CXCL9, and CXCL10, resulting in increased T-cell trafficking to the tumor (26). Furthermore, in patients treated with dacarbazine, CCL5, CXCL9, and CXCL10 expression was increased in chemotherapy-sensitive tumors, which was associated with increased T-cell infiltrate and enhanced patient survival (26). Other chemotherapeutic agents, such as dacarbazine, carboplatin, anthracycline and taxane, were also seen to increase the secretion of these chemokines and enhanced T cell trafficking to tumors (27, 45) (**Figure 1D**).

In preclinical mouse models, poor T-cell trafficking was associated with low levels of chemokine expression, such as ligands for CXCR3 and CXCR4 (73). Pre-treatment with oxaliplatin and cyclophosphamide induced a pro-inflammatory tumor microenvironment and enhanced secretion of CCL5, CXCL9, CXCL10, and CXCL16 by tumor-infiltrating macrophages, leading to enhanced ROR1-CAR-T cell infiltration (60). The combination was also able to increase CD8^+^ T cell infiltration and improved tumor control (47). This immunomodulatory effect was not restricted to oxaliplatin, other platinum-based drugs, such as carboplatin, have also increased tumor CD8^+^ T cell populations and enhanced CCL5 and CXCL10 mRNA levels in lung cancer cells (46).

Taken together, certain chemotherapeutic drugs can alter the immunosuppressive tumor microenvironment to facilitate CAR-T cell trafficking, infiltration, expansion, and anti-tumor efficacy in solid tumors. However, the evidence listed above also indicated the choice of chemotherapeutic drugs, the dosage and the treatment schedule must be taken into serious consideration when designed in combination with CAR-T cell therapy.

## 3 Challenges for the combination of chemotherapy with CAR-T cell therapy

Chemotherapy is well known for its cytotoxic effects, which may also have a deleterious effect on CAR-T cell viability and function, inducing apoptosis of immune cells, and often leading to lymphopenia (6). The cytotoxic effect of chemotherapy is often mediated *via* mechanisms such as DNA damage and cell cycle arrest, and preferentially targets rapidly dividing cells such as tumour cells and bone marrow stem cells. The effect is drug and dosage dependent. Some drugs, such as cyclophosphamide and fludarabine, are considered as non-myeloablative but lymphodepleting. They may affect the transferred CAR-T cells both in circulation and at tumor site. Thus, the systemic delivery of chemotherapy may not only affect the endogenous T cell population generated at bone marrow, but also induces cytotoxicity on transferred CAR-T cells directly. Because CAR-T cells are usually given in one dose or several doses in a short period of time, the long persistence of the CAR-T cells and sustained anti-tumor function are the keys to therapeutic success. Thus, any adjuvant treatment post-CAR-T infusion may have an impact on long-term CAR-T cell efficacy.

After chemotherapy regimens, T cell numbers decrease, which gradually recovers over time (74). Notably, chemotherapy may preferentially deplete certain T-cell populations over others. In glioblastoma patients treated with combined radiotherapy and temozolomide, an increased effector memory T-cell (T_EM_) population and a decreased CD45RA-expressing effector memory T-cell population (T_EMRA_) population was observed (75). Additionally, cyclophosphamide treatment was found to decrease the mitochondrial function of naïve T-cells significantly more than central memory (T_CM_) and effector memory (T_EM_) T cells (76). The evidence indicated that certain chemotherapeutic drugs might significantly impair the CAR-T cell function when used in combination.

Chemotherapy may also negatively regulate the CD4^+^ T-cell population. Compared with CD8^+^ T cells, naïve CD4^+^ T cells did not recover to pre-treatment numbers in breast cancer patients following chemotherapy (77). Another study found that naïve CD4^+^ T cells were preferentially depleted after chemotherapy, with memory CD4^+^ T cells forming the majority of the CD4^+^ T cell population instead (78). This preferential depletion of naïve CD4^+^ T cells may negatively affect CAR-T cell efficacy, given the importance of CD4^+^ T cells in achieving long-term remission (79).

In CAR-T cell therapy, less differentiated T-cell subsets, including naïve T-cells and T_CM_ cells, have shown greater efficacy compared to more differentiated effector T-cell and T_EM_ cells (80). Thus, the susceptibility of naïve T-cells towards chemotherapy may decrease CAR-T cell efficacy when the chemotherapy is used as an adjuvant treatment.

Besides the effect on T-cell subsets, chemotherapy, such as carboplatin and taxane, may also induce the expression of immune checkpoint molecules on T cells, including PD-1 and CTLA4 in breast cancer patients, as a feedback immunosuppressive pathway following immune activation (45). This effect was also seen in other drugs, such as the oxaliplatin plus cyclophosphamide combination (47). Although combining with checkpoint blockades such as anti-PD1 or anti-CTLA4 may overcome this issue, the potential chemotherapy-induced early T-cell exhaustion should be considered for combination treatments.

Despite these challenges, the heterogenous response between studies highlights the multitude of factors affecting chemotherapy efficacy in combination with CAR-T therapy, including the type of drugs, treatment dose, regimen schedule, and cancer types. Thus, a successful combination of chemotherapy and CAR-T cell therapy relies greatly on the design of the treatment. To achieve this, the treatment plan must be optimised to allow long-term CAR-T cell persistence and anti-tumor function to provide the best clinical outcomes. In addition, CAR-T cells can also be engineered to resist these drugs, making it easier to optimise this combination. Such engineering may take inspirations from the chemo-resistance mechanisms observed in cancers, such as the upregulation of detoxifying molecules in response to platinum-based chemotherapies (81). The engineered expression chemo-resistance proteins such as platinum transporters and chelators may confer CAR-T cell resistance against platinum-based drugs. The CRISPR drug screening technology can also be used to select potential targets for drug resistance in CAR-T cells. Such candidates can then be conditionally expressed in CAR-T cells to gain chemo-resistance. In addition, chemotherapeutic drugs targeting DNA damage and the cell cycle also induce apoptosis in CAR-T cells. Thus, engineering mutated apoptotic machinery, such as the BCL-2 family proteins, may facilitate resistance to this drug-induced apoptosis in CAR-T cells. However, engineering CAR-T cells to make them resistant to apoptosis should be viewed with caution to avoid unexpected uncontrolled CAR-T cell proliferation.

Moreover, clinical strategies for combining chemotherapy with CAR-T cell therapy need to be evaluated. This combination therapy could be performed *via* two approaches. Chemotherapy’s anti-tumor effect and immunomodulatory properties are traditionally studied in the context of specific tumor types. Thus, chemotherapeutic drugs could be selected based on the patient’s tumor type. However, given the significant heterogeneity between tumor subtypes, such as in breast cancer, different drugs may be needed for different patients with the same cancer (82). Alternatively, a more personalised approach where drug choices are tailored to the patient’s TME may be used. By testing patient samples, the TME components can be revealed. This information can be used to select the best chemotherapeutic drugs to facilitate the CAR-T cell attack. For instance, if poor T cell trafficking is due to immunosuppressive cells such as MDSCs and Tregs, cyclophosphamide may be used. If the immune exclusion is caused by CAFs, paclitaxel could be used instead. In addition, it should be noted that many chemotherapeutic drugs, including paclitaxel, have a wide range of effects on the TME, e.g., depleting MDSCs, Tregs and CAFs at the same time, as outlined in **Table 1**. These personalised strategies focus more on the challenges for CAR-T cells in each individual patient’s tumor microenvironment and will be more likely to achieve better clinical outcomes for the ‘chemo plus CAR-T’ combination therapy.

## 4 Conclusion

Although a limited number of clinical trials have been performed, the combination treatment of chemotherapy and CAR-T cell therapy has significant capacity to improve the current clinical outcomes. It is a promising option for patients with advanced solid tumors, and further studies on the dose, treatment schedule, immune context, tumor types, and CAR-T cell engineering should be investigated to achieve best clinical outcomes. In addition, more mechanistic studies are still needed to understand how these therapies will best work together. Our increased understanding of the immunomodulatory effects of chemotherapy, together with the engineering of novel CAR-T cells, will further facilitate this combination strategy in the clinic and benefit more cancer patients.

## Author contributions

Writing-original draft preparation, AW, XO. Writing-review and editing, CD’S, PN, JZ. Supervision, PN. AW and XO share first authorship. JZ, PN and CD’S share senior authorship. All authors contributed to the article and approved the submitted version.
